# Mechanistic Insights into the Multiple Functions of Niacinamide: Therapeutic Implications and Cosmeceutical Applications in Functional Skincare Products

**DOI:** 10.3390/antiox13040425

**Published:** 2024-03-30

**Authors:** Cíntia Marques, Farid Hadjab, Alexandre Porcello, Kelly Lourenço, Corinne Scaletta, Philippe Abdel-Sayed, Nathalie Hirt-Burri, Lee Ann Applegate, Alexis Laurent

**Affiliations:** 1 Development Department, LOUNA REGENERATIVE SA, CH-1207 Geneva, Switzerland; c.marques@louna-aesthetics.com (C.M.); a.porcello@louna-aesthetics.com (A.P.); k.lourenco@louna-aesthetics.com (K.L.); 2 Development Department, Albomed GmbH, D-90592 Schwarzenbruck, Germany; f.hadjab@albomed.eu; 3 Regenerative Therapy Unit, Lausanne University Hospital, University of Lausanne, CH-1066 Epalinges, Switzerland; corinne.scaletta@chuv.ch (C.S.); philippe.abdel-sayed@chuv.ch (P.A.-S.); nathalie.burri@chuv.ch (N.H.-B.); 4 STI School of Engineering, Federal Polytechnic School of Lausanne, CH-1015 Lausanne, Switzerland; 5 Center for Applied Biotechnology and Molecular Medicine, University of Zurich, CH-8057 Zurich, Switzerland; 6 Oxford OSCAR Suzhou Center, Oxford University, Suzhou 215123, China; 7 Manufacturing Department, LAM Biotechnologies SA, CH-1066 Epalinges, Switzerland; 8 Manufacturing Department, TEC-PHARMA SA, CH-1038 Bercher, Switzerland

**Keywords:** antioxidants, cellular respiration, cosmeceuticals, dermal fillers, formulation, functional ingredients, hyaluronic acid, niacinamide, skincare, viscoelastics

## Abstract

Niacinamide (or nicotinamide) is a small-molecule hydrosoluble vitamin with essential metabolic functions in mammalian cells. Niacinamide has become a key functional ingredient in diverse skincare products and cosmetics. This vitamin plays a pivotal role in NAD^+^ synthesis, notably contributing to redox reactions and energy production in cutaneous cells. Via diversified biochemical mechanisms, niacinamide is also known to influence human DNA repair and cellular stress responses. Based on decades of safe use in cosmetics, niacinamide recently gained widespread popularity as an active ingredient which aligns with the “Kligman standards” in skincare. From a therapeutic standpoint, the intrinsic properties of niacinamide may be applied to managing acne vulgaris, melasma, and psoriasis. From a cosmeceutical standpoint, niacinamide has been widely leveraged as a multipurpose antiaging ingredient. Therein, it was shown to significantly reduce cutaneous oxidative stress, inflammation, and pigmentation. Overall, through multimodal mechanisms, niacinamide may be considered to partially prevent and/or reverse several biophysical changes associated with skin aging. The present narrative review provides multifactorial insights into the mechanisms of niacinamide’s therapeutic and cosmeceutical functions. The ingredient’s evolving role in skincare was critically appraised, with a strong focus on the biochemical mechanisms at play. Finally, novel indications and potential applications of niacinamide in dermal fillers and alternative injectable formulations were prospectively explored.

## 1. Introduction

**Figure 1:**
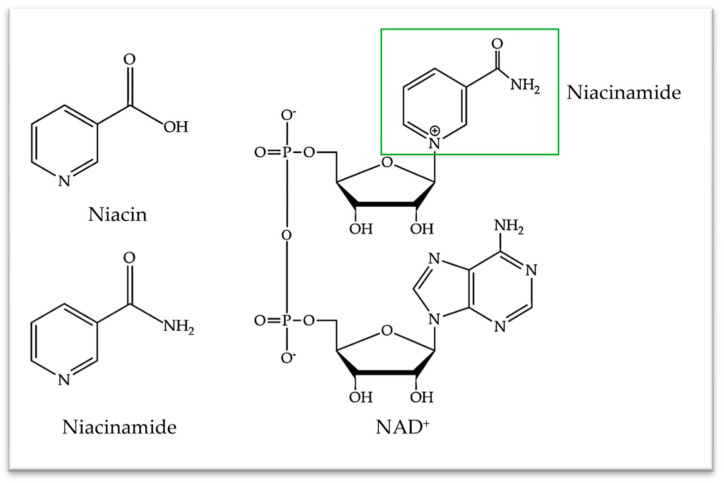
Molecular structures of niacin and niacinamide in the vitamin B3 complex and their molecular constitutive role in NAD^+^ synthesis. NAD^+^, nicotinamide adenine dinucleotide.

Niacinamide, a member of the vitamin B3 family, has emerged as an important and polyvalent functional ingredient in topical skincare products and cosmetics. Consisting of two hydrosoluble molecular forms (i.e., niacin or nicotinic acid and niacinamide or nicotinamide; Figure 1), this vitamin is essential to various mammalian cell physiological processes.

However, these chemical entities are not stored in the human body and their main dietary forms consist of niacinamide, niacin, and tryptophan from foodstuff sources (Figure 1) [1]. Of note, the assimilated niacin is bio-converted into niacinamide in vivo [1]. Importantly, while niacin and niacinamide share identical vitamin functions, their pharmacological and toxicological profiles diverge [2]. Therein, the adverse effects of high enteral doses of niacin include cutaneous flushing (i.e., especially of the face and neck) and pruritus. In contrast, niacinamide does not possess cutaneous vasodilating properties, being extremely well-tolerated by the skin (e.g., no irritation, redness, burn, sting, or itch issues) [2,3]. Thus, niacinamide is a preferred formulation option for cosmetic applications.

From a cellular functional standpoint, niacinamide is crucial for the synthesis of nicotinamide adenine dinucleotide (NAD^+^; Figure 1), which is then used to synthesize NADH and NADPH coenzymes. The latter are essential coenzymes in redox reactions and energy generation processes within mammalian cells [4,5,6]. Fundamentally, a documented involvement in DNA repair and stress responses may explain niacinamide’s role in cellular longevity and health improvement. Such effects are notably deployed through the attenuation of oxidative stress and inflammatory responses [6,7,8]. Furthermore, niacinamide has been clinically systemically used to treat several diseases such as pellagra, schizophrenia, and type I diabetes [9,10]. It has also demonstrated intrinsic anti-inflammatory potential in osteoarthritis [11]. 

Labeled as a “GRAS” (i.e., generally recognized as safe) food additive and nutrient, niacinamide was granted approval for cosmetic use in Japan and the European Union [12]. Therein, due to its potent antioxidant activity, niacinamide has been widely used as an excipient in various types of dermo-cosmetic formulations [13]. Of note, clinical tests on niacinamide found no stinging with concentrations up to 10%, no irritation up to 5%, and no irritancy during a 21-day test at 5% concentration [12]. Over the last decade, niacinamide has become highly popular as an active ingredient, since it very closely meets the “Kligman standards” of cosmeceutical formulation analysis [14,15]. Namely, the following elements have been shown:

(i) Niacinamide effectively penetrates the stratum corneum, reaching its intended target in sufficient amounts;
(ii) It exerts its effects via specific biochemical mechanisms of action within cutaneous cells and on human skin;
(iii) Peer-reviewed, double-blinded, placebo-controlled clinical trials with statistically significant results substantiating the efficacy claims have been published.

In terms of topical applications, niacinamide has been widely employed for the treatment of acne vulgaris, melasma, atopic dermatitis, rosacea, and psoriasis, while its oral administration for non-melanoma skin cancer prevention has also been studied [10,16]. Notably, niacinamide’s popularity is also attributed to its recognized skin-antiaging properties. Importantly, cutaneous aging involves causative factor-dependent morphological changes, encompassing epidermal thinning (i.e., especially in chronologically aged skin), wrinkles, laxity, dermal elastosis (i.e., especially in photoaging), telangiectasia, and aberrant pigmentation [17]. The underlying mechanisms are complex and may involve cellular senescence, DNA damage, oxidative stress, inflammation, and genetic mutations, which can be mitigated or reversed by niacinamide. Indeed, this vitamin was proven to be effective in clinical studies for fine lines/wrinkles, hyperpigmentation spots, yellowing, rough texture, and red blotchiness [3]. Moreover, this specific functional ingredient is one of the most widely used antioxidants in anti-aging topical formulations, with an increase in usage of about 10% between 2013 and 2018 [18]. Specifically, several comprehensive reviews, such as those by Boo et al. (2021), Poljsak and Milisav (2018), or Levin and Momin (2010), provide detailed summaries of niacinamide’s efficacy in clinical studies [6,7,14].

Generally, the present narrative review delves into the less-known mechanisms of action of niacinamide in cosmetic formulations. Specifically, it explores the effects of this polyvalent ingredient on NAD^+^-dependent enzymes and its functions as an antioxidant, its anti-inflammatory and antimicrobial activities, with a special focus on its role in cutaneous anti-aging. Additionally, critical discussion points and perspective elements were set forth herein around current formulation challenges and potential novel applications of niacinamide (e.g., in dermal fillers and cutaneous bio-stimulants). Overall, the present work provides concise and specific insights into niacinamide’s important and evolving role in functional skincare and cutaneous appearance optimization.

## 2. Mechanistic Insights into the Functions and Activities of Niacinamide

### 2.1. NAD^+^-Dependent Enzyme Regulation by Niacinamide

Being essential to NAD^+^ formation (Figure 1), niacinamide influences the activity of several enzymes that are critical to basic cellular activity, such as sirtuins and poly-(ADP-ribose) polymerases (PARP). Notably, sirtuins are a family of proteins, dependent on and stimulated by NAD^+^ formation, but which are inhibited by an excess of free niacinamide [19,20]. Thus, exogeneous administration of niacinamide is considered to inhibit SIRT-1 activity [21,22]. Similarly, niacinamide-mediated inhibition of SIRT-2 was correlated with decreases in melanoma tumoral cell growth [23].

Importantly, these metabolic proteins are involved in several skin-relevant cellular functions and processes, including aging, UV damage responses, oxidative stress, inflammation, and wound repair [22,24,25]. Research has also revealed the role of sirtuins in a variety of skin diseases, including melanoma and non-melanoma skin cancers [23,24,26]. Thus, sirtuins are important as conserved regulators of cutaneous aging and longevity [19,27]. However, given that the sirtuin family consists of various proteins, which have been identified in different cell types and tissues, the ultimate and net effects of their inhibition or stimulation may vary [24].

Generally, since sirtuins and PARP are both part of basic cellular biochemical pathways, their functions and effects are intertwined. For example, by inhibiting SIRT-1, niacinamide increases PARP-1 activity, which deploys an anti-inflammatory action [28]. Therefore, niacinamide can also be classified as a PARP-1 agonist [28]. In addition to decreasing the expression level of pro-inflammatory cytokines, the PARP-1 enzyme plays an important role in DNA repair [29]. As mentioned before, PARP activity depends on NAD^+^ availability, meaning that DNA repair will also be modulated by niacinamide bioavailability [5,30,31]. Notably, in vitro studies demonstrated that niacinamide could effectively reverse DNA damage, decrease keratinocyte death rates, and prevent cellular senescence [30,32].

Furthermore, PARP-1 plays a fundamental role in DNA repair and in the regulation of genes involved in inflammation, apoptosis, and cellular differentiation [29]. Interestingly, both PARP-1 hyperactivation and inhibition can be associated with cell death. Therein, niacinamide contributes to maintaining an equilibrium in enzymatic activity and in cell homeostasis through PARP-1 regulation [29,33]. Several additional in vitro studies with keratinocyte models showed that niacinamide effectively promoted and reversed DNA damage, even when these were caused by extrinsic factors (e.g., UV exposure) [34,35,36].

Overall, the competitive interplay for NAD^+^ between PARP and sirtuins allows to conjointly set forth multiple theories of cutaneous aging [19]. Thus, a better understanding of their respective actions could help to further unravel aging mechanisms. Notwithstanding, as a coenzyme in the glycolysis pathway, niacinamide is an essential component to ensure efficient DNA repair by avoiding cellular senescence, apoptosis, and carcinogenesis, through its roles in PARP and sirtuin regulation [24,25].

### 2.2. Antioxidant Activity of Niacinamide

Oxidative stress (i.e., generation of reactive oxygen species [ROS] and free radicals) occurs naturally due to internal metabolic dysfunctions and is intensified by external damaging factors (e.g., UV radiation, pollution) [37,38]. This physiological cellular state impairs DNA repair, increases pro-inflammatory cytokine synthesis, and stimulates matrix metallopeptidase (MMP) production, contributing to skin aging and skin diseases [29,37]. Therefore, oxidative stress is usually considered the core driving factor in cutaneous aging [17].

**Figure 2:**
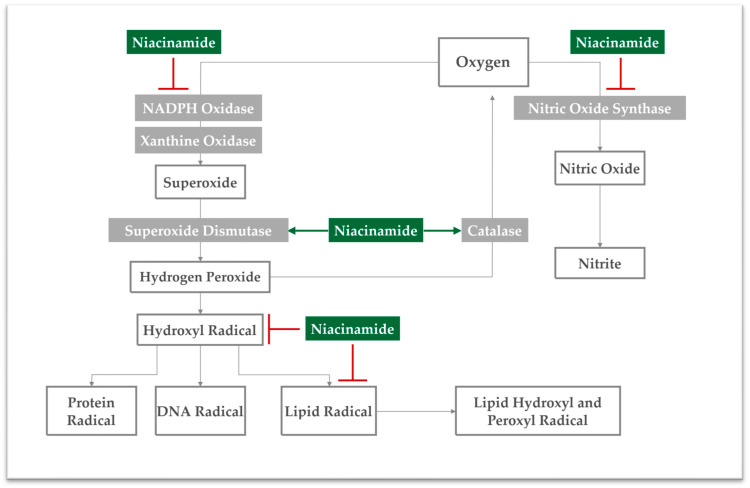
Mechanistic overview of the intracellular antioxidant mechanisms of niacinamide within the ROS cascade. The key enzymes involved in the cascade are depicted in grey boxes. The inhibitory effect of niacinamide on these enzymes is illustrated in red. Conversely, the stimulatory effect of niacinamide is represented by green arrows.

As reviewed by Nakai et al., oxidative stress results from an increase in ROS and other oxidants, exceeding the cellular antioxidant capacity [39]. Therein, ROS and free radicals are produced within a complex pathway, which involves several enzymes such as NADPH oxidase and nitric oxide synthase (NOS) [39]. Specifically, these two enzymes play an important role in the intracellular oxidation pathway, as they intervene in the initial steps of the ROS cascade (Figure 2) [39].

As mentioned before, niacinamide is a precursor for NAD^+^, which is essential to produce NADP^+^ [6,30]. Niacinamide has been associated with lower NADP/NADPH ratios, thus its administration might decrease NADPH oxidase expression and activity. Thereby, niacinamide contributes to decreased superoxide radical concentrations, as demonstrated before in keratinocyte cultures [30,34]. This specific function is supported by another in vitro study with keratinocytes, in which nicotinamide administration restored NAD reserves, thereby completely reversing ROS accumulation [30]. Importantly, niacinamide was also shown to increase superoxide dismutase levels, which also contributes to the decrease in superoxide radical concentrations [40].

At the same time, niacinamide is well-known to interfere with nitric oxide synthase (NOS) activity, but the exact mechanism remains unclear. Some in vitro studies with macrophages have shown that niacinamide indirectly inhibits NOS activity by allowing nitric oxide (NO) to inhibit its own formation [41,42]. In other words, NO will activate a cascade that ultimately inhibits NOS [41]. Other evidence suggests that niacinamide can also inhibit NOS mRNA production in cells, thereby decreasing the enzyme’s activity and NO formation [43]. Furthermore, decreased NOS activity also derives from NAD’s inhibition of PARP, which leads to a reduction in NOS expression and consequently to a decrease in NO synthesis [44]. 

Besides regulating NADPH oxidase and NOS action, niacinamide was also demonstrated to increase the enzymatic activity of catalase, transforming hydrogen peroxide back into oxygen and water. As a result, decreased formation of hydroxyl radicals may be observed, as well as inhibition of dermal collagen fibril disruption by MMPs [45,46]. Conjointly with enzymatic regulation attributes, niacinamide presents intrinsic scavenger activity, being able to directly neutralize ROS and free radicals. Therein, high scavenger activity was recorded for hydroxyl radicals and low scavenger activity was recorded for NO [31,47]. Generally, by normalizing the activity of antioxidant enzymes and by neutralizing oxidants, niacinamide was shown in vivo and ex vivo to ultimately prevent protein oxidation and lipid peroxidation (Figure 2) [5,13,34,45,48,49]. Thus, it was shown that the antioxidant functions of niacinamide safeguard cellular membrane integrity against oxidation [13]. Finally, this functional ingredient is widely used as an antioxidant in anti-aging topical formulations, which are mainly composed of 4% to 5% niacinamide [6,18].

### 2.3. Anti-Inflammatory Activity of Niacinamide

Niacinamide is well-known for its anti-inflammatory properties. It is widely used to combat inflammatory acne, with proven clinical effectiveness [50,51,52]. Furthermore, niacinamide was shown to reduce inflammation in a metabolic syndrome model, in inflammatory bowel disease, osteoarthritis, Alzheimer’s disease, and in models of nociceptive and inflammatory pain [11,49,53,54,55]. Of note, inflammatory responses are complex phenomena, also exacerbated by oxidative stress, which increases inflammatory cytokine release [37]. As previously discussed, niacinamide presents a strong antioxidant activity, which ultimately contributes to decreased inflammatory responses. 

In addition to reducing ROS levels, niacinamide is also known to inhibit the secretion of pro-inflammatory cytokines through PARP regulation. Macrophage treatment with niacinamide resulted in inhibited SIRT, which consequently increased PARP-1 activity [28]. Importantly, PARP-1 activation is linked to COX-2 inhibition and to increased B-cell lymphoma-6 protein (BCL6) expression, with anti-inflammatory effects [28]. Furthermore, COX-2 inhibition by niacinamide was also associated with a decrease in PGE2 secretion by activated macrophages, since it is a major COX-2-dependent prostaglandin [56].

Notwithstanding, niacinamide is also known to directly inhibit PARP, leading to a reduction in NOS expression and consequently to a decrease in NO synthesis [44,57]. Additionally, PARP inhibition by niacinamide also decreases PGE2 secretion and myeloperoxidase activity, which also contributes to its anti-inflammatory action [57]. Even though the effect of niacinamide on PARP receptors requires further studies to be completely understood, it clearly leads to an overarching and multimodal anti-inflammatory action.

From a more specific mechanistic viewpoint, niacinamide inhibits the production of pro-inflammatory cytokines (e.g., TNF-α, PGE2, IL-1, IL-6, and IL-8) by controlling NFκB-mediated transcription and increases the production of anti-inflammatory mediators (e.g., IL-10 and MRC-1) [28,32,54,56,58,59]. The decrease in IL-1aRA/IL-1a inflammatory skin biomarkers was also observed in a clinical study involving 40 panelists after two weeks of administration of a 5% niacinamide emulsion [60,61,62]. Notably, the decrease in cytokine responses upon niacinamide administration is considered to be dose-dependent [56,58]. Additionally, niacinamide also contributes to the suppression of the expression of MHC class II, through the reduction in interferon-γ levels. This was achieved both with in vitro fibroblast cultures and in vivo, with a downregulation of the immune response [40,60]. Moreover, by decreasing the levels of inflammatory mediators, niacinamide prevents keratinocyte senescence, consequently decreasing the production of senescence-associated secretory phenotype (SASP) [32]. Of note, senescent cells remain metabolically active and secrete several molecules, including pro-inflammatory cytokines, chemokines, and proteases [61]. A study by Bierman et al. showed that by preventing keratinocyte senescence, niacinamide also decreases the production of cytokines associated with SASP [62]. 

Niacinamide’s anti-inflammatory action also stabilizes mast cells (i.e., in the dermis), suppressing mast cell degranulation and anaphylactic responses in mice [63,64]. Mast cell activation would lead to fibroblast senescence, wherein niacinamide operates on various components of the inflammatory cascade. Thereby, the vitamin actively participates in the restoration of a cellular equilibrium [65]. Furthermore, since pruritus inflammatory symptoms are thought to be related to the release of histamine from cutaneous mast cells, niacinamide is considered to possess an anti-pruritic activity [66]. Moreover, the symptoms are also potentiated by dry skin, which is attenuated by the stimulation of ceramide synthesis by niacinamide. Therefore, the described combination of mast cell stabilization and preservation of the skin barrier places niacinamide as a key soothing molecule during pruritic outbreaks.

### 2.4. Antimicrobial Activity of Niacinamide

Niacinamide was shown to possess antibacterial, antifungal, and antiviral activity, with the literature focusing mainly on its antibacterial activity. The latter was demonstrated in murine Gram-positive and Gram-negative sepsis models, as well as against *Escherichia coli* and *Staphylococcus aureus* [67,68]. Niacinamide’s antimicrobial activity was also shown to effectively prevent biofilm formation in several clinical studies [69,70,71]. Moreover, niacinamide’s antibacterial activity strategically places it as an interesting active ingredient for acne treatment, as sustained by several clinical studies [72,73,74]. Therein, its topical application for acne vulgaris treatment displayed efficacy levels similar to those of clindamycin (i.e., in terms of anti-inflammatory activity). Furthermore, it is underlined that niacinamide possesses antimicrobial activity against *Cutibacterium acnes* [71]. Interestingly, niacinamide was more efficacious in oily skin types than in non-oily skin, with concentrations ranging from 2% to 4% in topical applications [50,72,73,74].

Since niacinamide’s direct action does not affect bacterial survival, its antimicrobial activity is mainly attributed to the stimulation of both neutrophil action and antimicrobial peptide (AMP) synthesis [68,75]. Additionally, niacinamide is known to activate multiple cellular pathways, which can collectively lead to protection from pathogens, along with innate immunity activation [68]. For example, niacinamide inhibits the nuclear PARP enzyme, thereby preventing the integration of proviral DNA during viral infection [63]. Furthermore, a synergistic antifungal activity of niacinamide and amphotericin B was demonstrated against *Candida albicans* and *Cryptococcus neoformans*, even though the mechanism remains unclear [69].

Importantly, AMPs are part of the skin’s innate immune system, having broad antibacterial activity against Gram-positive and negative bacteria and also showing antifungal and antiviral activity [67,76,77]. In fact, an increase in AMPs upon niacinamide application has been shown to protect gut epithelial cells from infection [78]. Especially, AMPs are also present in human skin tissues, including psoriasin (S100A7), RNase, lysozyme, cathelidicins, and defensins [7,76,77]. It has been specifically demonstrated that sebocytes, keratinocytes, and neutrophils can produce defensins, while the last two can also synthesize cathelidicins [76].

Studies demonstrated that keratinocytes treated with a niacinamide solution were successfully stimulated to synthesize AMPs in vitro, providing protection against skin infection [68]. Furthermore, there is evidence that niacinamide can potentiate the activity of cathelidicin LL-37 [77,79]. Specifically, due to niacinamide’s amphiphilic nature, the molecule modulates the physical properties of the bacterial membrane and increases LL-37 bioavailability [77,79]. Of note, several studies correlated niacinamide’s antimicrobial activity with increased neutrophil activity, demonstrating that niacinamide selectively enhanced the neutrophil killing of *Staphylococcus aureus* and *Citrobacter rodentium* [75,77,80].

Of note, the antimicrobial activity of neutrophils consists of the combination of three different actions: phagocytosis, NETosys, and degranulation [81]. During the degranulation process, several molecules are released, including AMPs. An in vitro study with lung epithelial cells infected with *Streptococcus pneumoniae* revealed that niacinamide blocks the SIRT-1 receptor, reducing defensin expression but increasing IL-8 levels [21]. This interleukin recruits neutrophils, which can then release AMPs, meaning that niacinamide might have a neutrophil-dependent effect on the increase in AMPs [78,82]. However, additional studies are needed to understand its influence on AMP release by alternative skin cell types.

### 2.5. Sebum Production Reduction Activity of Niacinamide

Acne is a multifactorial disease, characterized by different pathways, which include excess sebum production, abnormal keratinization, bacterial colonization by *Cutibacterium acnes*, and inflammation [4,83,84]. As described above, niacinamide possesses anti-inflammatory and antibacterial activities, thereby contributing to the dual management of important acne symptoms.

As concerns sebaceous activity, several clinical studies report that preparations with 2% to 5% niacinamide can effectively reduce sebum production following topical application, notably in Asian and Caucasian populations [74,85,86,87,88]. However, the mechanism which leads to the sebostatic action of niacinamide remains unknown. Interestingly, niacin (Figure 1), which is part of the vitamin B3 family, was shown to activate HCA2 receptors, which are known to regulate sebum production in human sebocytes. Specifically, niacin’s interaction with HCA2 receptors induces a transient Ca^2+^ elevation, which culminates with lower sebum production [89]. 

Importantly, niacin’s carboxylic moiety is essential to bind to the receptor, whereas niacinamide contains an amide group instead. Therefore, niacinamide does not bind to the HCA2 receptor, yet one can argue that niacinamide can be bio-converted to niacin, thereby indirectly decreasing sebum production [6,90]. While clinical studies widely support the benefits of niacinamide for sebum reduction, more studies are needed to understand the underlying mechanisms.

### 2.6. Skin Anti-Yellowing Activity of Niacinamide

Glycation is a spontaneous oxidative cross-linking reaction that occurs between proteins (e.g., dermal collagens) and endogenous sugars. This reaction leads to the formation of advanced glycation end products (AGEs), which affect different structures and physiological functions of the skin [91,92]. Glycation is part of the physiological skin aging process, notably leading to the formation of a yellow end-product. Specifically, this compound accumulates in the cutaneous extracellular matrix due to its long biological half-life, leading to a yellowing appearance [3,91,93].

Importantly, oxidative radicals are the most important contributors to the glycation process. Thus, antioxidants play an essential role in preventing AGE formation [91]. Due to its potent antioxidant activity, niacinamide dampens this natural phenomenon, decreasing sallowness (i.e., yellowing) of the skin, with proven efficacy in clinical studies enrolling Caucasian females [3,93,94].

### 2.7. Skin Lightening Activity of Niacinamide

Lightening agents can improve hyperpigmentation disorders caused by hypermelanosis (i.e., increased deposition of melanin), such as melasma, axillary hyperpigmentation, lentigo senilis, and post-inflammatory hyperpigmentation [95,96]. Furthermore, uneven skin pigmentation is one of the major changes characterizing extrinsic aging, which increases the demand for compounds with depigmenting activity [97]. 

Importantly, niacinamide has been successfully applied in clinical studies for the treatment of hyperpigmentation. In one study, a 4% niacinamide formulation successfully decreased axillary hyperpigmentation [98]. Therein, the treatment induced a significant colorimetric improvement, which was associated with its antimelanogenic action [98]. Of note, in axillary hyperpigmentation, inflammation is also considered to be an associated condition. Niacinamide effectively decreased epidermal melanin (i.e., decreased melanin expression) and inflammatory marker levels (i.e., decrease in the number of mononuclear and phagocytic cell infiltrates) [98]. Furthermore, niacinamide’s role in decreasing skin pigmentation and inflammatory infiltrates was also correlated with its effectiveness in the treatment of melasma [96]. Therein, in comparison with hydroquinone (i.e., traditionally used for melasma treatment), niacinamide’s clinical efficacy took longer to be demonstrated, but the latter caused fewer adverse effects, making it a safer option for longer treatments [96]. 

As concerns niacinamide’s antimelanogenic action, studies have revealed that the molecule does not affect tyrosinase catalytic activity or melanogenesis in melanocytes, meaning that niacinamide does not seem to influence the synthesis of melanin [99,100]. Notwithstanding, visible pigmentation in mammals requires the transfer of melanin granules from melanocytes to keratinocytes. In this case, there is strong evidence that niacinamide blocks the transfer of melanosomes from melanocytes to surrounding keratinocytes [95,99,101]. Thus, niacinamide has an antimelanogenic action, which is dose-dependent and reversible, yet the exact inhibitory mechanism of melanosome transfer requires further exploration [101].

Of note, keratinocytes produce melanotrophic factors that affect melanocyte proliferation, dendricity, and melanin synthesis [102]. Therein, niacinamide reduces the secretion level of IL-6 by keratinocytes (i.e., a melanotrophic factor) [99,103]. Moreover, melanocytes express PGE2 receptors, which respond to PGE2 secretion by keratinocytes, leading to filopodia formation and driving melanosome transfer to keratinocytes [102]. Therein, niacinamide can reduce PGE2 production by keratinocytes, so one could consider this as a mechanism to prevent melanosome transfer from melanocytes to surrounding keratinocytes [62]. However, in vitro experiments are required to test this hypothesis. Finally, melanosome transfer by melanocytes is also stimulated by AGEs. Specifically, as niacinamide is also known to reduce AGE production, this mechanism may also contribute to reducing cutaneous hyperpigmentation [3,92].

### 2.8. Cutaneous Extracellular Matrix and Skin Barrier Enhancement by Niacinamide

In proximity to the dermal–epidermal junction, the dermis is mostly composed of fibroblasts and their secretory proteins, which form the extracellular matrix [17]. Cutaneous aging leads to a natural decrease in fibroblast activity, with several studies showing a decrease in SIRT expression [24]. Consequently, a decrease in ECM protein levels (e.g., collagen and elastin) is observed, which is linked to both natural aging and photoaging [104]. Therein, the reduction in protein synthesis is also correlated to a ROS increase, which induces cell senescence and degradation of ECM components, mediating premature skin aging [104]. Therefore, the intrinsic antioxidant activity of niacinamide contributes to cellular homeostasis, thereby contributing to skin ECM integrity. Additionally, niacinamide stabilizes mast cells (i.e., in the dermis) by several mechanisms, such as through the inhibition of inflammatory cytokine synthesis and through NAD^+^ increases [63,64]. Since mast cell degranulation leads to fibroblast senescence, and since niacinamide prevents this phenomenon, the latter contributes to normal fibroblast activity maintenance [65].

Fibroblasts also secrete matrix metallopeptidases (MMP), a group of catabolic enzymes that lead to collagen degradation [105]. Importantly, MMP synthesis is stimulated by ROS and inflammatory cytokines. Thus, niacinamide inhibits MMPs, due to its antioxidant and anti-inflammatory activities [17,106,107]. Furthermore, it was shown that niacinamide inhibits elastase activity, preserving the integrity of elastin in cutaneous ECM [107]. Interestingly, niacinamide was also shown to boost collagen (and procollagen) production by fibroblasts in ex vivo murine studies and in human trials. Therein, the follow-up demonstrated a discontinuous improvement of collagen IV expression in the basal membrane by niacinamide [8,98,106,107]. Moreover, fibroblast secretion of elastin and fibrillin was also increased by niacinamide administration [106,107].

In addition to collagen destruction, collagen glycation also negatively impacts ECM molecular organization, disrupting the longitudinal ordering of collagen fibrils, which affects cell adhesion and migration [108]. As previously discussed, the antioxidant action of niacinamide prevents collagen glycation, thereby contributing to cutaneous ECM integrity. Keratinocytes are also essential to ECM homeostasis, and niacinamide benefits their biological activity. 

Of further note, nicotinamide boosts ceramide synthesis by activating the mRNA expression of serine palmitoyl transferase (i.e., key enzyme for sphingolipid synthesis) and by accelerating keratinocyte differentiation [63,109,110]. Niacinamide prevents keratinocyte senescence due to photoaging and oxidative stress, with a study showing that it promotes the repair of DNA damage induced by UV rays [32,35,36]. Moreover, niacinamide boosts the expression of a differentiated type of keratin K1 [110]. However, the molecule has no effect on the proliferation of keratinocytes [99]. In summary, niacinamide’s action affects both fibroblasts and keratinocytes, improving ECM quality and skin barrier integrity, for an overall increase in cutaneous health.

### 2.9. Cutaneous Anti-Aging Activity of Niacinamide

Skin aging corresponds to a series of physiological changes observed in cutaneous tissues including thinning or thickening, dryness, laxity, dermal elastosis, telangiectasia, aberrant hyperpigmentation, and the development of wrinkles [17,24,111]. These can be caused by several internal factors (e.g., natural aging) or through contact with external factors such as ozone, particulate matter, cigarette smoke, ultraviolet radiation (i.e., photoaging), and an unhealthy lifestyle, which accelerates skin aging [17,37,39,112]. Of note, the apparent signs of cutaneous aging may be different based on the main causative factor. Therein, dermal elastosis and epidermal thickening are considered to be hallmarks of photoaging, whereas epidermal thinning is characteristic of chronological skin aging.

Photoaging is one of the most discussed processes in the literature, since solar exposure leads to chronic inflammation and oxidative stress, accelerating premature aging. At the molecular level, the sunlight deteriorates DNA strands, with the cell damage being amplified by the concomitant oxidative stress. Ultimately, this induces cellular senescence, meaning that there is a decrease in the production of proteins (i.e., collagen/elastin) and other critical components of skin structure [104]. Additionally, there is an increase in the secretion of proinflammatory cytokines, chemokines, and proteases, which accelerates ECM degradation. When stimulated by UV radiation, niacinamide increased the activity of enzymes involved in cellular metabolism or energy production and significantly protected against immune suppression caused by UVBs or longwave UVAs [113]. Therefore, the photoprotective effects of niacinamide can also help to explain its nonmelanoma and melanoma preventive actions [23,24,26].

Of note, dermal fibroblast and keratinocyte bio-stimulation is a common target in antiaging treatments, aiming to prevent or reverse cellular senescence and to re-establish ECM integrity. Specifically, niacinamide was proven to prevent cellular senescence, including in keratinocyte and fibroblast models [30,32,62,65]. As mentioned before, the anti-senescence activity of niacinamide derives primarily from its anti-inflammatory actions, such as the resulting decrease in inflammatory mediators and the inhibition of mast cell degranulation [32,62,65]. Moreover, the accumulation of DNA damages induced by oxidative stress also leads to cell senescence, which can be reversed by niacinamide, as shown by several studies [30,32,36]. Finally, fibroblast and keratinocyte senescence is associated with a general decrease in collagen, elastin, and keratin production. Importantly, several studies have demonstrated niacinamide’s efficacy for stimulating collagen, elastin, and ceramides production [63,98,106,107,109,110]. Thus, niacinamide may tangibly improve cutaneous ECM quality by acting at a cellular level, thereby reversing apparent aging signs.

**Figure 3:**
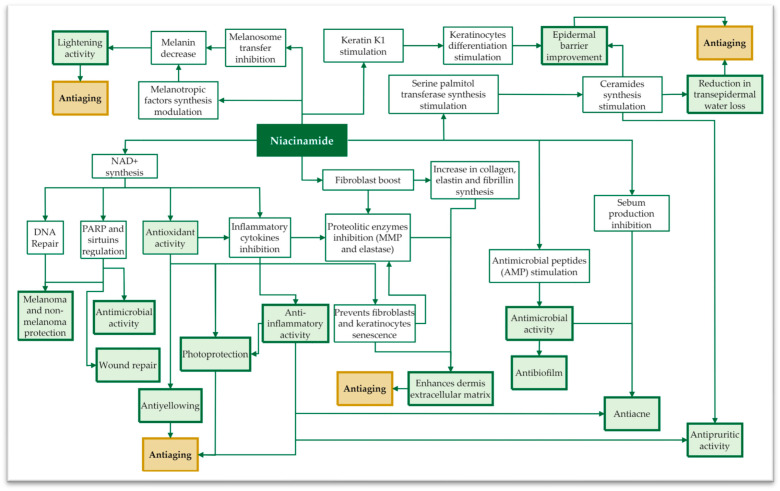
Overview of key cutaneous processes influenced by niacinamide and the resulting biological functions. The diagram provides a concise overview of niacinamide’s multifaceted impact on skin health and its role in mitigating the observable aging process.

As discussed previously, niacinamide is a precursor of NAD^+^; therefore, it prevents the depletion of cellular energy and regulates the redox status of the cell. Additionally, it balances the levels of various cellular metabolites, maintaining cellular homeostasis [6,63]. By acting as a potent antioxidant, niacinamide tackles oxidative stress, which is frequently considered the core driving force in cutaneous aging (i.e., due to both intrinsic and extrinsic factors) [17,39]. In conclusion, the anti-aging action of niacinamide may not be considered as a specific feature, but as a combined result of its overall activity (Figure 3). 

Due to its influence on NAD^+^-dependent enzyme regulation and its anti-yellowing, lightening, antioxidant, and anti-inflammatory activities, niacinamide preserves the extracellular matrix and contributes to the re-establishment of cutaneous functionality, preventing premature aging [62].

## 3. Niacinamide Formulation Challenges and Opportunities

As mentioned throughout this review, the vast majority of niacinamide’s action is exerted in the epidermis or dermis. However, a majority of the considered niacinamide-based formulations (i.e., as available on the market) are for topical application. Consequently, such formulations need to be able to achieve transdermal delivery in order to exploit the full potential of niacinamide’s activity. Of note, transdermal drug delivery is challenging since molecules need to permeate the stratum corneum (i.e., the outermost layer of the skin), which serves as a main barrier to drug penetration.

On one hand, the stratum corneum layer is mainly composed of dead, keratinized cells with an intercellular matrix consisting mainly of ceramides, cholesterol, and free fatty acids [114]. On the other hand, niacinamide is a class I drug (i.e., in the Biopharmaceutics Classification System) with high aqueous solubility and permeability, which is challenging for transdermal delivery [115,116]. Therefore, niacinamide permeation and deposition are highly dependent on specific permeation enhancers, with hydrophobic excipients increasing niacinamide retention in the upper skin layers (e.g., by encapsulating niacinamide into oily vesicles) [115,116,117,118]. 

A study comparing three different oil-in-water niacinamide formulations concluded that skin-barrier-mimetic formulations were more efficient in increasing niacinamide deposits, despite lower initial niacinamide concentrations [116]. Another study reported a successfully increased niacinamide deposition while decreasing permeation (i.e., low systemic distribution), by encapsulating the molecule into oily vesicles, which were then suspended in a hydrogel for topical application [118]. 

More recently, there has been an effort to develop alternative means to increase niacinamide transdermal delivery. For example, niacinamide extrudates (i.e., prepared by hot melt extrusion) were also shown to have increased skin deposition attributes in comparison to a gel form, due to its higher adhesivity [119]. Furthermore, microneedles formulated with sodium hyaluronate and amylopectin were shown to efficiently encapsulate and deliver niacinamide, while niacinamide-imprinted starch-based biomaterials effectively treated skin hyperpigmentation [120,121].

Another convenient way to circumvent the transdermal delivery challenge is to directly inject niacinamide into the dermis, such as with the NCTF 135 HA^®^ product (Laboratories FillMed, Paris, France), a liquid mesotherapy formulation designed for intradermal microinjection [122]. Besides this specific commercial formulation, the authors have no knowledge of alternative intradermal niacinamide forms approved for cosmetic human use in Europe. While NCTF 135 HA^®^ does contain the molecule, mesotherapy formulations are known to contain ingredients in “minute doses”; therefore, intradermal injections of higher niacinamide concentrations would require additional safety assessments [123]. Outside of cosmetic applications, niacinamide injectables are available to treat vitamin deficiencies (e.g., Infuvite^®^, FDA-approved) and as an absorption modifier in a fast-acting subcutaneous insulin formulation (i.e., Fiasp^®^, EMA- and FDA-approved) [124]. Therefore, niacinamide’s safety is in all probability maintained even when injecting higher doses for cosmetic purposes.

In parallel to low-viscosity liquid formulations, niacinamide can also be found in the Innoryos^®^ formulation (Albomed, Schwarzenbruck, Germany), a hyaluronan-based hydrogel for osteoarthritis viscosupplementation [125]. Therein, the vitamin not only stabilizes the hydrogel but also displays an anti-inflammatory action in osteoarthritic joints. Of note, cross-linked hyaluronic acid (HA) dermal fillers are among the most popular injectable formulations in cosmetics, due to their documented quality, efficacy, and reversibility [126]. These have also been efficiently loaded with different molecules, such as proline and glycine, upgrading cross-linked HA dermal fillers into drug delivery platforms [127]. 

**Figure 4:**
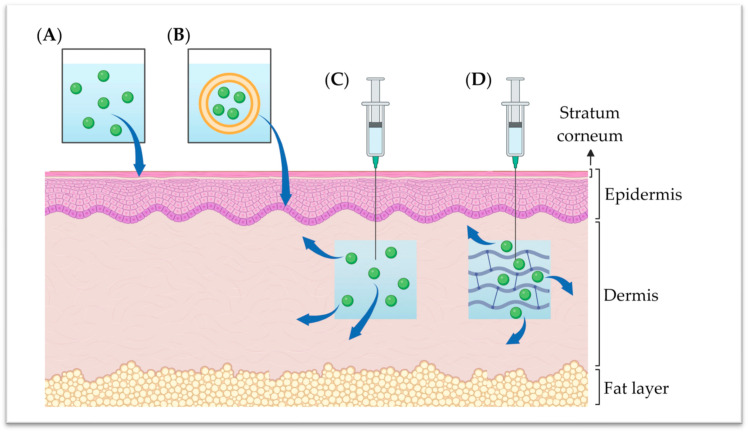
Niacinamide formulation variants and their permeation/administration into the skin. The aqueous formulation (**A**) is highly limited as regards penetration in the stratum corneum. However, an oil-in-water formulation (**B**) is characterized by enhanced niacinamide permeation into the epidermis. For a more direct impact on the dermis, aqueous solution (**C**) and hydrogel (**D**) intradermal injections could offer increased niacinamide bioavailability and effectiveness.

Considering the successful commercialization of Innoryos^®^, one could tangibly design HA-based dermal fillers loaded with niacinamide. Specifically, this combination would potentially enhance filler product stability, thereby extending its residency time, an issue commonly encountered in aesthetic procedures [125]. Moreover, the incorporation of niacinamide in HA fillers could facilitate the vitamin’s action on the dermis, amplifying its beneficial effects. Importantly, recent research has shown that crosslinked HA-based hydrogels could efficiently load and release niacinamide, which places these dermal fillers as promising vehicles for future administration modalities of niacinamide (Figure 4) [128].

## 4. Conclusions

Niacinamide, a member of the vitamin B3 family, is a key ingredient in cosmetics. Well-tolerated by the skin, niacinamide is essential for NAD^+^ synthesis or PARP and sirtuin regulation. By acting on the fundamental biochemical reactions within the cell, it displays potent antioxidant properties and anti-inflammatory activities. Moreover, studies have confirmed niacinamide’s antimicrobial activity, making it effective against bacteria and preventing biofilm formation. With proven efficacy in acne treatment and sebum reduction, niacinamide also addresses cutaneous hyperpigmentation and glycation-related yellowing. Additionally, it contributes to the skin’s extracellular matrix integrity by preserving collagen, inhibiting matrix-degrading enzymes, or promoting collagen and elastin production. Overall, niacinamide tackles several major issues related to skin aging, positioning itself as a multipurpose functional ingredient in skin rejuvenation. Despite transdermal delivery challenges, new formulations (e.g., skin barrier-mimetic bases) and new administration routes (i.e., intradermal injection) are promising to improve future cosmeceutical applications of niacinamide.

## Data Availability

Not applicable.

## Abbreviations

AGE	Advanced glycation end products
AMP	Antimicrobial peptide
BCL6	B-cell lymphoma-6 protein
DNA	Deoxyribonucleic acid
ECM	Extracellular matrix
EMA	European Medicines Agency
FDA	US Food and Drug Administration
GRAS	Generally recognized as safe
HA	Hyaluronic acid
HCA2	Hydroxycarboxylic acid receptor 2
IL	Interleukins
MHC	Major histocompatibility complex
MMP	Matrix metalloproteinases
MRC-1	Mannose receptor C-1
mRNA	Messenger ribonucleic acid
NAD^+^	Nicotinamide adenine dinucleotide (oxidized)
NADH	Nicotinamide adenine dinucleotide (reduced)
NADPH	Nicotinamide adenine dinucleotide phosphate
NO	Nitric oxide
NOS	Nitric oxide synthase
PARP	Poly-(ADP-ribose) polymerase
PGE2	Prostaglandin E_2_
ROS	Reactive oxygen species
SASP	Senescence-associated secretory phenotype
SIRT	Sirtuin
TNF-α Tumour necrosis factor alpha	
US	United States of America
UV	Ultraviolet
